# Supplementary material for: Waste Separation in Cafeterias: A Study among University Students in the Netherlands
Source: Int J Environ Res Public Health. 2018 Dec 31;16(1):93. doi: 10.3390/ijerph16010093 (PMC6338995; doi:10.3390/ijerph16010093)
Supplement: Supplementary file 1 [file ijerph-16-00093-s001.zip › data/maastrichtuniversity.eu.qualtrics.pdf]

Intro

This survey is a part of my Master thesis in Human Decision Science at Maastricht University, School of Business and Economics. As you may be aware of, the school has implemented a new waste sorting system in the buildings of the University. You now have the option to separate paper, plastic and general waste. This survey will explore your experience with the waste separation system.

The study should take approximately 5-10 minutes to complete. Your responses will be anonymous and no identifying information will be collected such as email addresses, IP addresses or names.

If you have any questions or comments about the survey feel free to contact me, Ágústa Dan Árnadóttir, at a.arnadottir@student.maastricht.nl. Your participation is greatly appreciated.

By pressing the orange button you confirm that you have been informed of the study and have read the written information. You have had the opportunity to ask questions about the study and you have been able to think about your participation in the study. You further confirm that you agree on the data gathered in the questionnaire being used for further analysis.

Please note that participation is voluntary and you can end the survey at any point.

Descriptives

Please state your gender

Male

Female

Other

Please state your age

Please indicate your status

Student at Maastricht University

Faculty / Personnel at Maastricht University

Other

What faculty of Maastricht University do you belong to?

Faculty of Arts and Social Sciences (FASoS)

Faculty of Health, Medicine and Life Sciences (FHML)

Faculty of Humanities and Sciences (FHS)

Faculty of Law

Faculty of Psychology and Neuroscience (FPN)

School of Business and Economics (SBE)

Knowledge

The next questions will show a picture of a product that can be purchased at the University caféteria. Please choose the appropriate trash bin for the products *packaging.*

Please drag the image to the appropriate trash bin.

Items

1 / 1

Plastic

Paper

1.

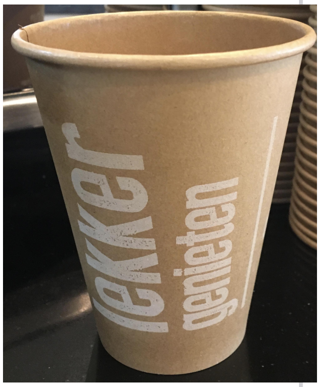

General waste / Rest

Please drag the image to the appropriate trash bin.

Items

2.

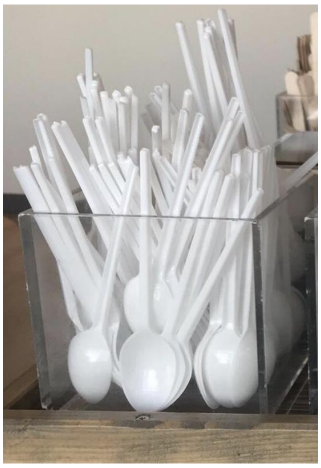

Plastic

Paper

General waste / Rest

Please drag the image to the appropriate trash bin.

Items

3.

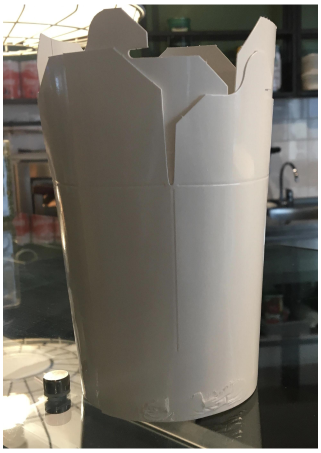

Plastic

Paper

General waste / Rest

Please drag the image to the appropriate trash bin.

Items

Plastic

Paper

4.

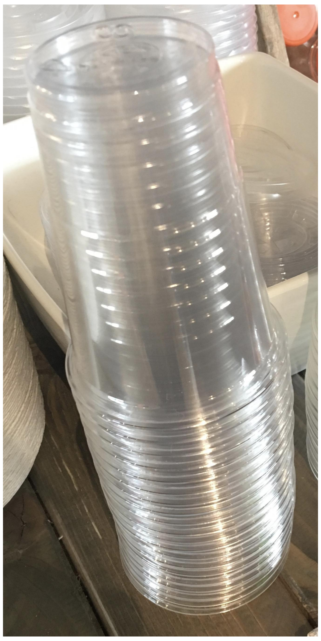

General waste / Rest

Please drag the image to the appropriate trash bin.

Items

5.

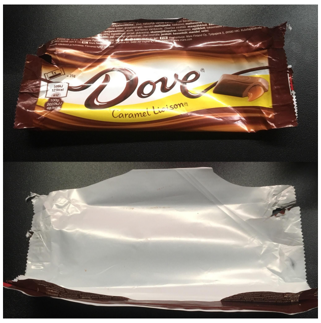

Plastic

Paper

General waste / Rest

Please drag the image to the appropriate trash bin.

Items

6.

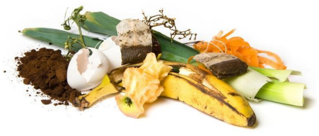

Plastic

Paper

General waste / Rest

Please drag the image to the appropriate trash bin.

Items

7.

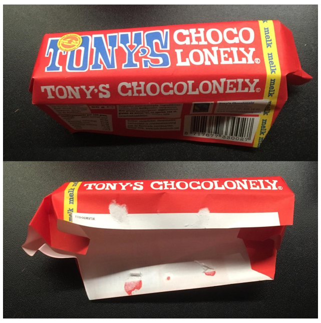

Plastic

Paper

General waste / Rest

Please drag the image to the appropriate trash bin.

Items

8.

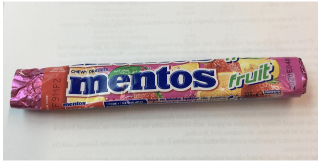

Plastic

Paper

General waste / Rest

Please drag the image to the appropriate trash bin.

Items

Plastic

9.

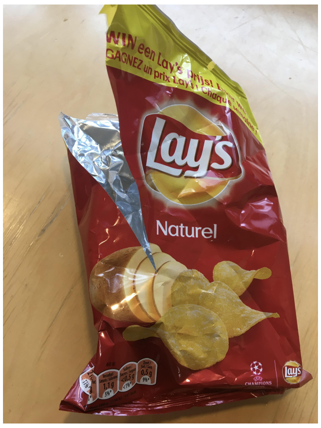

Paper

General waste / Rest

Please drag the image to the appropriate trash bin.

Items

10.

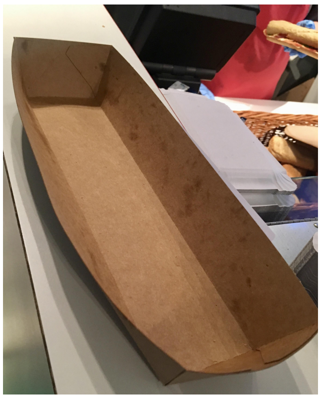

Plastic

Paper

General waste / Rest

Please drag the image to the appropriate trash bin.

Items

11.

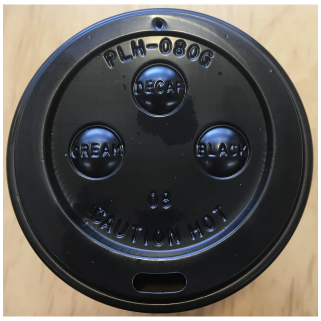

Plastic

Paper

General waste / Rest

Please drag the image to the appropriate trash bin.

Items

12.

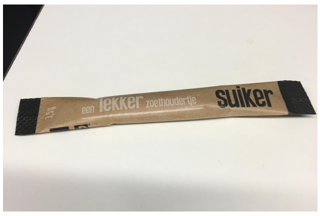

Plastic

Paper

General waste / Rest

Please drag the image to the appropriate trash bin.

Items

13.

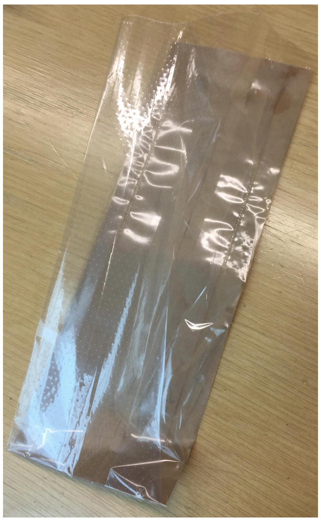

Plastic

Paper

General waste / Rest

Please drag the image to the appropriate trash bin.

Items

14.

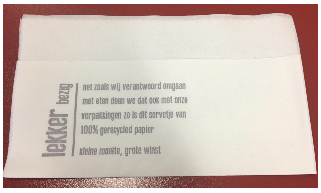

Plastic

Paper

General waste / Rest

Please drag the image to the appropriate trash bin.

Items

15.

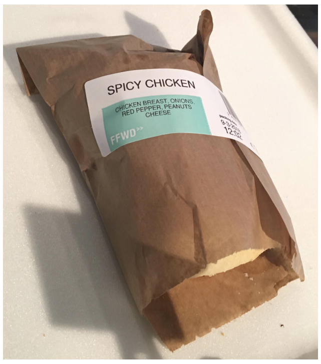

Plastic

Paper

General waste / Rest

Correct answers

Not everybody is aware of the schools guidelines on how to sort waste in Maastricht University. Therefore, here below you can see how the products you have sorted previously, among others, are supposed to be sorted. This is in accordance with UM guidelines and has been approved by the UM Green office.

Please inspect these guidelines carefully.

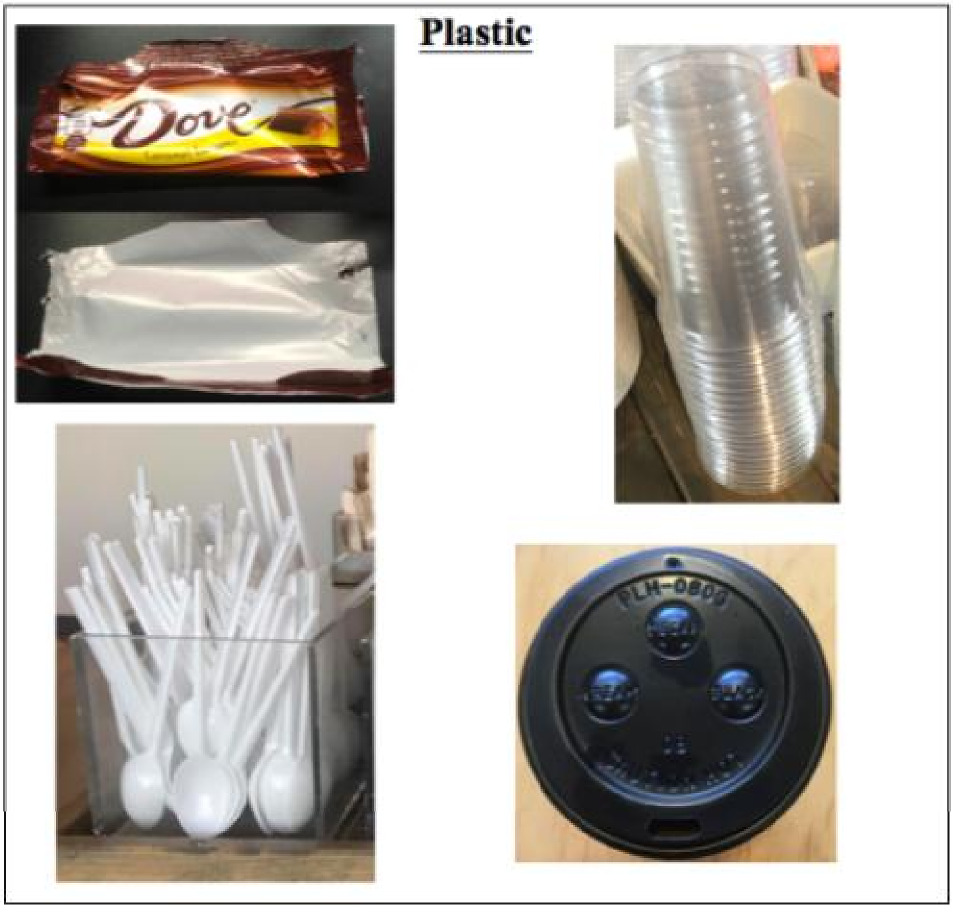

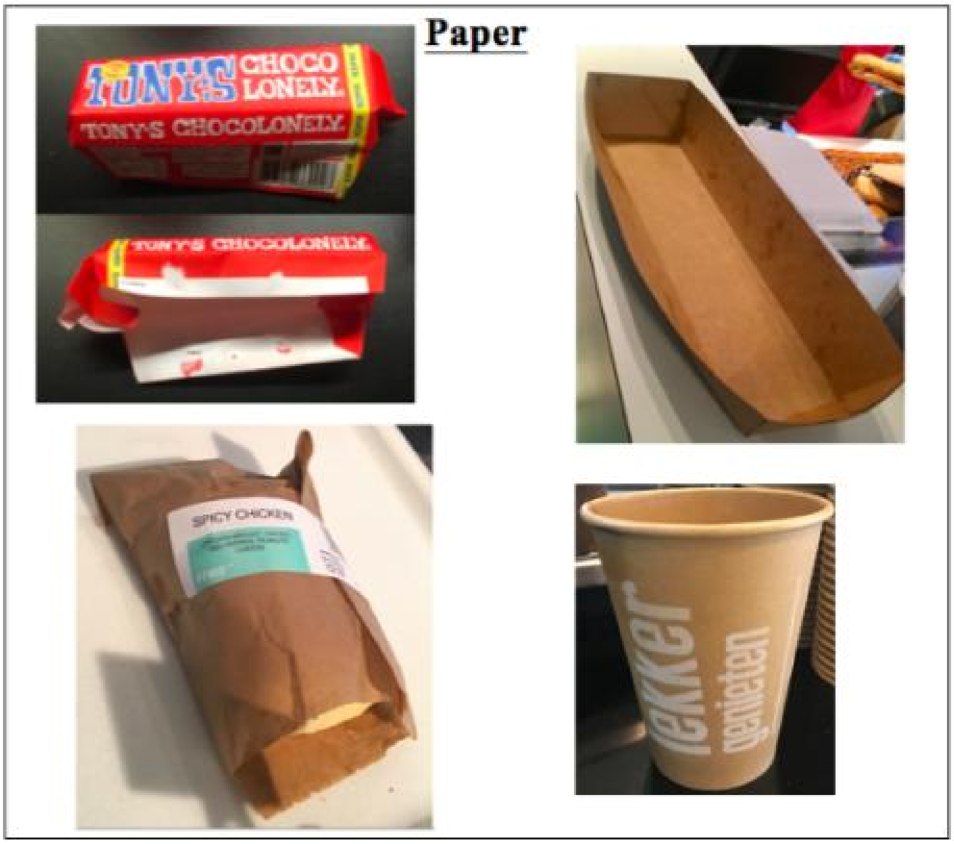
Paper
TONY'S CHOCO LONELY
TONY'S CHOCOLONELY
SPICY CHICKEN
lekker
genieten

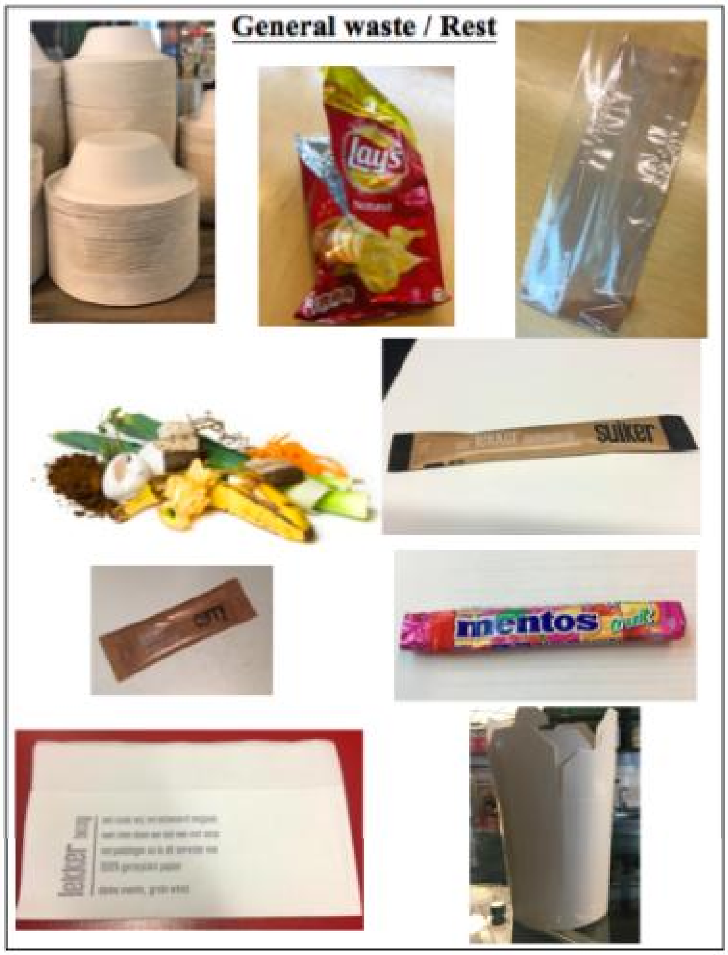
General waste / Rest
Lay's
suiker
mentos

TPB questionnaire

Here below are a few questions regarding your waste separation during lunch breaks for the next 3 months.

Please answer the following questions by indicating on a scale of 1 to 7 how much each statement applies to you.

Me separating my waste accurately during lunch breaks for the next 3 months is

	1	2	3	4	5	6	7	
Good	O	O	O	O	O	O	O	Bad

Me separating my waste accurately during lunch breaks for the next 3 months is

	1	2	3	4	5	6	7	
Pleasant	O	O	O	O	O	O	O	Unpleasant

Most people who are important to me think that I should separate my waste accurately during lunch breaks for the next 3 months

	1	2	3	4	5	6	7	
True	O	O	O	O	O	O	O	False

Most people at Maastricht University separate their waste accurately during lunch breaks for the next 3 months

	1	2	3	4	5	6	7	
Agree	O	O	O	O	O	O	O	Disagree

I am confident that I can separate my waste accurately during lunch breaks for the next 3 months

	1	2	3	4	5	6	7	
True	○	○	○	○	○	○	○	False

Separating my waste accurately during lunch breaks for the next 3 months is

Easy	○	○	○	○	○	○	○	Difficult

If answer for previous question is 5 or higher, why is it difficult?

If I really wanted to, I could separate my waste during lunch breaks for the next 3 months

	1	2	3	4	5	6	7	
Likely	○	○	○	○	○	○	○	Unlikely

I intend to separate my waste accurately during lunch breaks for the next 3 months

	1	2	3	4	5	6	7	
Definetly do	○	○	○	○	○	○	○	Definetly do not

I expect to separate my waste accurately during lunch breaks for the next 3 months

	1	2	3	4	5	6	7	
Likely	○	○	○	○	○	○	○	Unlikely

In the past 3 months how often have you separated your waste accurately during lunch breaks?

	1	2	3	5	6	7	
Almost always	○	○	○	○	○	○	Never

Name at least one thing that prevents you from separating your waste accurately during lunch breaks for the next 3 months.

How would you like to receive information from the green office about changes in waste sorting guidlines?

Email

Social Media

Flyers

Posters

Other

Powered by Qualtrics
